# Gamma Delta T Cell Therapy for Cancer: It Is Good to be Local

**DOI:** 10.3389/fimmu.2018.01305

**Published:** 2018-06-08

**Authors:** C. David Pauza, Mei-Ling Liou, Tyler Lahusen, Lingzhi Xiao, Rena G. Lapidus, Cristiana Cairo, Haishan Li

**Affiliations:** ^1^American Gene Technologies International Inc., Rockville, MD, United States; ^2^Department of Medicine, Marlene and Stewart Greenebaum Comprehensive Cancer Center, University of Maryland School of Medicine, Baltimore, MD, United States; ^3^Institute of Human Virology, Department of Medicine, University of Maryland School of Medicine, Baltimore, MD, United States

**Keywords:** gamma delta, T cell, cancer, immuno-oncology, Vdelta2 gamma delta T cells, targeted immunotherapy

## Abstract

Human gamma delta T cells have extraordinary properties including the capacity for tumor cell killing. The major gamma delta T cell subset in human beings is designated Vγ9Vδ2 and is activated by intermediates of isoprenoid biosynthesis or aminobisphosphonate inhibitors of farnesyldiphosphate synthase. Activated cells are potent for killing a broad range of tumor cells and demonstrated the capacity for tumor reduction in murine xenotransplant tumor models. Translating these findings to the clinic produced promising initial results but greater potency is needed. Here, we review the literature on gamma delta T cells in cancer therapy with emphasis on the Vγ9Vδ2 T cell subset. Our goal was to examine obstacles preventing effective Vγ9Vδ2 T cell therapy and strategies for overcoming them. We focus on the potential for local activation of Vγ9Vδ2 T cells within the tumor environment to increase potency and achieve objective responses during cancer therapy. The gamma delta T cells and especially the Vγ9Vδ2 T cell subset, have the potential to overcome many problems in cancer therapy especially for tumors with no known treatment, lacking tumor-specific antigens for targeting by antibodies and CAR-T, or unresponsive to immune checkpoint inhibitors. Translation of amazing work from many laboratories studying gamma delta T cells is needed to fulfill the promise of effective and safe cancer immunotherapy.

## Introduction

Human T cells expressing the Vγ9JγPVδ2 T cell receptor [also designated Vγ2Jγ1.2Vδ2 (1, 2)] comprise 1–5% of circulating lymphocytes in healthy adults. Treating peripheral blood mononuclear cells (PBMC) with phosphorylated intermediates from the isoprenoid biosynthesis pathway [isopentenyl-pyrophosphate (IPP) and (E)-4-hydroxy-3-methylbut-2-enyl pyrophosphate (HMBPP)] (3–5) or aminobisphosphonate inhibitors of farnesyl diphosphate synthase (FDPS) (6) stimulate proliferation, cytokine secretion, and cytotoxic effector function of Vγ9Vδ2 T cells. The Vγ9Vδ2 T cell response to phosphorylated compounds (phosphoantigens) or aminobisphosphonate is almost exclusive to Vδ2 cells with a JγP rearrangement and responding cells frequently use public Vγ9JγP sequences that are shared widely in the population [reviewed in Ref. (7)]. Because the Vγ9Vδ2 T cell response to phosphoantigen or aminobisphosphonate is ubiquitous in the healthy human population and the expanded cells have potent effector functions, many investigators are developing immunotherapies based on selective activation of the Vγ9Vδ2 T cell subset. Increasingly the focus of γδ T cell research is on cancer immunotherapy. Here, we review oncology applications for this important component of natural tumor surveillance and discuss obstacles to clinical translation of our basic knowledge about γδ T cells.

Circulating Vγ9Vδ2 T cells are diverse due to length and sequence variation in the CDR3 regions of both γ and δ chains but their uniform activation by phosphoantigen or aminobisphosphonate gives the appearance of an innate response. Similarly, the circulating adult Vγ9Vδ2 T cell repertoire is shaped by constant positive selection due to the presence of ubiquitous phosphoantigens produced by host cells or resident microbes. Chronic positive selection increases the proportion of Vγ9Vδ2 T cells in blood and reduces population diversity due to amplification of T cell clones mostly expressing public Vγ9JγP chains. With a TCR repertoire altered by chronic positive selection, human and non-human primates along with a few other species (8) maintain a pool of Vγ9Vδ2 T cells that is dominated by central and effector memory phenotype, contains 1 of every 40 memory T cells in the body, and reacts to appropriate stimuli with the speed and uniformity of innate immunity. These features, particularly the uniform response to common stimuli without MHC restriction, make the Vγ9Vδ2 T cell subset especially attractive for a variety of therapeutic applications in man.

In earlier studies of human γδ T cells it was discovered that several tumor cell lines directly stimulated the Vγ9Vδ2 subset without exogeneous phosphoantigen or aminobisphosphonate addition. A good example is the Daudi B cell line, which is a selective activator of Vγ9Vδ2 T cell proliferation and effector function (9). The Daudi cell line is unusual because it does not express β_2_-microglobulin, hence fails to MHC class I-related surface glycoproteins. However, other cell lines with MHC class I expression (10) are nonetheless capable of activating Vγ9Vδ2 T cells and of being targets for cytotoxicity. Using Daudi B cells to stimulate PBMC lead to expansion of multiple Vγ9Vδ2 T cell clones from healthy individuals and the pattern of responses was similar to the pattern of clonal expansion after IPP stimulation (11).

Multiple agents can be combined to achieve potent stimulation of Vγ9Vδ2 T cells. The best-known examples of phosphoantigen, aminobisphosphonate, or stimulatory cell lines were described above. In those examples, elevated levels of cellular IPP were critical to cell stimulation and IPP levels must be elevated in a cell that also expresses butyrophilin 3A1 on its surface (12–16). The butyrophilin 3A1 is only one part of a complex regulator of VγVδ2 T cell activation and is found within heteromeric complexes that control T cell activation (17). Evidence from physical studies supports a view that phosphoantigen binding to the cytoplasmic B30.2 domain of butyrophilin 3A1 induces a unique conformational change that propagates throughout the molecule (18–20). The idea that conformational change in butyrophilin 3A1 governs recognition by Vγ9Vδ2 T cells is consistent with positive selection of the Vγ9JγP rearrangement; JγP is the longest J segment and length seems to be crucial for TCR recognition (7, 11). The literature on butyrophilin and γδ T cell activation has been reviewed recently (18).

A requirement for the combination of IPP plus the human butyrophilin complex explains the lack of xenogeneic stimulation by non-human tumor cells despite their common production of IPP. Variations of this theme help to explain why cells infected with bacteria that are themselves capable of producing phosphoantigens similar to IPP (21–23) will stimulate Vγ9Vδ2 T cells. Combinations of T cell receptor cross-linking *via* antibody treatment plus cytokine or toll-like receptor agonists also stimulate Vγ9Vδ2 T cell proliferation and cytokine production (24).

The signals required to maximize cytotoxic effector activity are less clear, though C-type lectin receptors are known to be important. The NK receptor NKG2D is a potent activator of cytotoxic effector function and is expressed on the majority of stimulated Vγ9Vδ2 T cells (25). A smaller sub-population expresses the inhibitory receptor NKG2A (26, 27), and both subsets may contain activated Vγ9Vδ2 T cells expressing the CD16 low affinity Fc receptor, and are capable of being activated by IgG bound to target cells (28).

## Strategies for γδ T Cells in Immuno-Oncology (I/O)

The challenges to developing a cancer therapy based on activating γδ T cells are exemplified in the history of intravesical *Bacille Calmette–Guerin* (BCG), a strain of *Mycobacterium bovis* used for treating bladder cancer. Epidemiology studies in the early twentieth century linked tuberculosis with lower cancer incidence and lead to the introduction of BCG as a cancer vaccine in 1935 [reviewed in Ref. (29)]. By the 1970s BCG was becoming accepted for bladder cancer therapy and remains in use for this disease. It was reported that BCG is a potent stimulator for Vγ9Vδ2 T cells (30) and activated cells kill bladder cancer cells *in vitro* (31). These findings suggested a direct relationship between Vγ9Vδ2 T cell activation by locally administered BCG and subsequent destruction of tumors by direct cytotoxicity. Around 40 years later we know that Vγ9Vδ2 T cells are found at higher levels in urine from bladder cancer patients treated with BCG (32) and successful treatment is associated with increased levels of intratumoral CD19 B cells along with CD4, CD8, and γδ T cells (33). Today, bladder cancer treatment is evolving with the introduction of new immunotherapies despite our poor understanding of immune response triggered by BCG *in situ*, the extent to which γδ T cells are important for these responses, and the mechanisms of action for tumor reduction. Could we have a better therapy for non-invasive BCG based on a clearer understanding of the immune response? The answer is uncertain, but it is likely that such studies would produce more sophisticated biomarkers, better prognosis, and personalized treatment for bladder cancer. This review of γδ T cells and cancer therapy seeks to identify similar gaps in our current understanding of γδ T cell immunotherapy that may slow progress to the development of treatment strategies and clinical products.

Most studies on human γδ T cells have concentrated on the Vγ9Vδ2 T cell subset from peripheral blood. This reflects in part, the ease of obtaining primary cells for laboratory studies and the ability to grow large numbers of Vγ9Vδ2 T cells from human PBMC. More importantly, this choice reflects the biology of Vγ9Vδ2 T cells where the TCR repertoire is shaped by strong, positive selection pressure that maintains a circulating, innate-like T cell population. Collective efforts in many laboratories created a detailed picture of the effector activities for Vγ9Vδ2 cells and outlined the road map for clinical applications.

Unique properties of circulating Vδ1 cells are less defined, although they have been studied for treatment of some neoplastic diseases (34, 35). The majority of Vδ1 cells comprise the intraepithelial lymphocyte population of mucosal epithelia where they react to signals of stress by producing abundant cytokines and chemokines that influence mononuclear cell infiltration into damaged, infected, or malignant epithelium (36–38). Recent progress in expanding Vδ1 cells from blood (39, 40) will undoubtedly increase our knowledge about this subset. The capacity for CD30+ Vδ1 T cells to produce IL-17A and create inflammatory microenvironment (41) suggests this T cell subset may promote or inhibit cancer progression depending on the cell type, location, state of disease, and other factors. Consequently, the role for Vδ1 cells remains unclear, especially in solid tumors, but these cells hold promise for treating a select group of malignancies including leukemia (35).

The Vδ3+ subset has also been considered for therapeutic use but less is known about stimulatory antigens or properties of these cells. The Vδ3+ subset has been implicated in the response to herpesvirus infections including cytomegalovirus and Epstein–Barr virus (42–44). Clinical studies correlated elevated baseline Vδ3 levels with fewer herpesvirus outbreaks after iatrogenic immune suppression such as that employed in the transplant setting (45). However, Vδ3 cells are relatively rare in blood and conditions for expanding these cells *ex vivo* are poorly defined. Cellular recognition of EBV- or CMV-infected cells has also been documented for Vδ1 or Vδ2 cells (42, 46) and in rare cases, the Vδ5+ subset also recognized herpesvirus-infected cells (44).

Our ability to define an I/O strategy based on the biology of γδ T cells is impacted by many factors including the limited information about how these cells participate in natural tumor surveillance. It is critical to decide whether a focus on the well-known Vγ9Vδ2 T cell subset offers more advantages compared to exploring tumor-infiltrating lymphocyte populations, and how can we balance the pro-tumor and anti-tumor roles for Vδ1 cells (47). Can we find unique properties of Vδ3 or other minor subsets that are compelling for cancer therapy? Finally, should we be looking for platform approaches to γδ T cell I/O or create unique approaches for each type of malignancy? Answers to these questions will help to define pathways for clinical development of γδ T cell immunotherapies.

## Is There a Role for Vγ9Vδ2 T Cells in I/O?

There are compelling arguments for I/O strategies based on activating Vγ9Vδ2 T cells. This subset is abundant in blood and cells can be expanded *ex vivo* with simple protocols. Cytotoxic killing of many tumor types is well documented for Vγ9Vδ2 T cells and the range of targets is broad. Furthermore, activation of Vγ9Vδ2 T cells can be accomplished *ex vivo* or *in vivo* through stimulation with mammalian or microbial phosphoantigens, one of several widely used aminobisphosphonate drugs, TCR-cross linking monoclonal antibodies, butyrophilin cross-linking antibodies, or exposure to stimulatory tumor cells. This highly flexible system provides many opportunities for matching Vγ9Vδ2 T cell stimulation with a specific tumor target and allows for realistic consideration of both passive immunotherapy with *ex vivo* expanded cells, and *in vivo* therapy using direct activation of the Vγ9Vδ2 T cell subset.

By contrast, the list of tumor cell targets for Vδ1 or Vδ3 cells are narrow, but may be expanded in the future, and there is a concern regarding the pro-inflammatory nature of Vδ1 cells because of their propensity to express the cytokines IL-17 or IL-4. For an immediate, near-term I/O program we and others [reviewed in Ref. (48–51)] tend toward focusing on the natural tumor surveillance activities of Vγ9Vδ2 T cells.

Despite our enthusiasm for tumor immunotherapy involving Vγ9Vδ2 T cells, there is always doubt regarding their practical utility for clinical cancer care. We might (and should) ask: if natural tumor immunity is important and potentially useful for oncologic medicine, why does this surveillance system fail to catch and prevent malignant disease in the first place? In other words, do tumors escape immune surveillance or is the demonstrated tumor cytotoxicity of γδ, NK, NKT, MAIT, and other innate-like cells an *in vitro* property with little relevance to practical problems in cancer care? We will overcome these concerns by demonstrating consistent potency, clearly defined mechanisms of action, and objective clinical responses to γδ T cell immunotherapy.

## Tumor Mechanisms for Immune Evasion

Multiple mechanisms have emerged to explain tumor evasion of MHC-restricted responses. The conventional paradigm argues that innate immune activation precedes activation of antigen-specific T cells, especially CD8+ subsets, and in turn leads to recruitment of effector T cells capable of destroying the tumor [reviewed in Ref. (52)]. Multiple mechanisms at each step may result in tumor escape from T cell surveillance. Tumors may have an immunosuppressive microenvironment that fails to activate innate responses. Tumors may have reduced immunogenicity due to lack of tumor-specific antigens or downregulation of MHC molecules. Tumors may also inhibit the activation of T cell effector mechanisms through overexpression of ligands for immune inhibitory costimulatory molecules (checkpoints). The latter area has received much attention lately due to licensure of therapeutic monoclonal antibodies that prevent receptor:ligand engagement of inhibitory costimulatory receptors and their ligands resulting in activation of tumor-specific T cell responses.

Clinical studies with antibodies that block inhibitory costimulation documented a substantial impact on specific subsets of malignant diseases. For example, Merck’s Keytruda product (monoclonal antibody against PD-1) for metastatic melanoma, non-small cell lung cancer, and tumors with high expression of PD-L1 has been highly successful in several clinical settings [reviewed in Ref. (53)]. Success with inhibiting PD-1/PD-L1 interactions by blocking PD-1 (Keytruda and Nivolumab) or PD-L1 (Atezolizumab, Avelumab, and Duvalumab), and blocking of CTLA-4 (Ipilimumab) has stimulated interest in blocking several other receptor/ligand interactions with similar mechanisms of action. The continuing development of new checkpoint inhibitors is especially important because tumor escape from anti-PD-L1 has already been observed and involved upregulation of other inhibitory costimulation molecules (54). Clinical experiences will elucidate mechanisms for escape from checkpoint inhibitor antibody effects and drive the development of new monoclonal antibody drugs alone or in combination with other antibodies or alternate therapeutic modalities.

Whether PD-1 or other checkpoint inhibitors enable tumors to escape Vγ9Vδ2 T cell surveillance is an open question. Expression of PD-1 was increased after Vγ9Vδ2 T cell stimulation but pre-treatment of PD-L1 Daudi tumor cells with zoledronic acid was sufficient to render them susceptible to Vγ9Vδ2 T cell killing irrespective of PD-L expression (55). Cytotoxicity was less potent when PD-L was present on tumor cells; some studies noted that tumor cell killing was reduced by up to fivefold in the presence of PD-L (56). Pennington’s group (57) identified a CD24−/CD28−/CD16+ subset of Vγ9Vδ2 T cells, representing about 10% of total Vγ9Vδ2 T cells in blood from healthy donors, that expressed CD57 and had the highest proportion of PD-1 cells (14%) among all Vγ9Vδ2 T cell subsets. Both CD57 and PD-1 are presumed markers of inactivated or “senescent” T cells that have lost the potential for proliferation (58). When double-positive cells accumulated at the tumor site, it was usually taken as a sign of failed tumor immunity (59). However, recent literature suggests that effector function and capacity for proliferation may be differentiated on the basis of CD57 expression. In both CD4 T cells (60) and NK cells (61), CD57 expression identified cells that are potent for cytotoxicity but lack the capacity for expressing immune-suppressing cytokines including IL-10 or IL-21 and will not proliferate in response to stimulation (60). If Vγ9Vδ2 or other γδ T cells also become potently cytotoxic without producing regulatory cytokines, PD-1 and possibly CD57 may be markers for tumor effector activity and not signs of a failed immune response.

As noted above, expression of PD-1 increased after phosphoantigen or aminobisphosphonate stimulation of Vγ9Vδ2 T cells (55) and we might infer from studies of NK and CD4 cytotoxic T cells that PD-1 and CD57 identify non-proliferating but potently cytotoxic cells that do not express suppressive cytokines. It is important to define the conditions for generating such cells, to determine their life span in tumors and to understand whether they contribute meaningfully to tumor reduction. Such studies may also guide decisions about future clinical trials proposing combinations of Vγ9Vδ2-based treatment and checkpoint inhibitor antibodies.

Because the population of Vγ9Vδ2 T cells responds almost uniformly to phosphoantigen or aminobisphosphonate stimulation, there will be rapid proliferation of stimulated cells and continuing production of the PD-1+/CD57+ subset. The majority of Vγ9Vδ2 cells will have little or no expression of PD-1 resulting in tumor killing that is not abrogated by PD-L1 or PD-L2 ligands (56). While the impact of PD-1 on Vγ9Vδ2 T cells is still being evaluated, we can make a provisional conclusion that this immune checkpoint is not an obstacle to Vγ9Vδ2 T cell tumor therapy although it may impact potency. Whether other immune checkpoint molecules may be more important is still under study. At this time, it appears that Vγ9Vδ2 cell therapy may differ from αβ T cell effector mechanisms in the extent to which they are impacted by immune checkpoints. If this view holds true, it will become an important argument for the uniqueness of Vγ9Vδ2 T cell immunotherapy.

Beyond immune checkpoint regulation, there are complex interactions among T cells, NK cells, dendritic cells, mesenchymal cells, myeloid-derived suppressor cells, and even neutrophils that dictate the tumor microenvironment and benefit or inhibit the capacity for effective tumor immunity. Recent comprehensive reviews addressed many of the key mechanisms for immune suppression including the functions for regulatory γδ T cells and effects of their cytokines on tumor killing; we refer the reader to these excellent publications (62–66). In addition to the immunosuppressive cytokines, pro-inflammatory cytokines including IL-17A often promote tumor growth and may be produced at higher levels when intratumoral T cells are dominated by the Vδ1 subset (41, 67). Normally, Vδ2 T cells dominate peripheral blood in healthy individuals and are >2-fold more abundant than circulating Vδ1 cells. A study including more than 200 melanoma patients treated with the checkpoint inhibitor antibody ipilimumab (targeting CTLA-4) showed that individuals with an inverted ratio of blood γδ cell subsets (Vδ1 > Vδ2) had lower overall survival, and poorer outcomes were significantly associated with decreasing Vδ2 T cell levels during ipilimumab therapy (68). A similar relationship between Vδ2 and Vδ1 cells was noted for rectal carcinoma (67).

Inversion of the Vδ2 ÷ Vδ1 T cell ratio in blood was also observed in HIV disease [reviewed in Ref. (69)], where inversion is due to quantitative depletion of Vδ2 cells and expansion of the Vδ1 subset, similar to the melanoma case cited above. In HIV disease, Vδ2 T cell depletion is due to multiple factors including direct toxicity of the viral envelope glycoprotein (70) and inadequate levels of IL-18 (71). Expansion of the Vδ1 subset was linked to damage of the intestinal epithelium and translocation of stimulatory bacterial products into blood (72, 73). It is of interest that the melanoma study, which included ipilimumab therapy (68) and HIV disease are both characterized by rising Vδ1 cells plus falling Vδ2 T cell levels. Notably, cancer is an important co-morbidity of HIV disease with rates greatly exceeding the general population along with increased susceptibility to a broad range of cancer types (74, 75). In future, we hope to apply knowledge from γδ T cell clinical cancer trials, to understanding and mitigating the increased cancer risk in HIV disease.

We do not yet understand why increased levels of Vδ1 T cells in blood is a risk factor for melanoma or HIV-associated cancer. This is due partly to the complex nature of the Vδ1 T cell subset. While Vδ1 from peripheral blood were cytotoxic for colon cancer cells (40), an IL-17A-producing subset of Vδ1 cells promoted tumor growth (76). Studies using peripheral blood may not reflect the properties of mucosal Vδ1 T cells. Overall, we will need to understand how to expand Vδ1 T cells *in vivo* or *ex vivo*, and to enrich beneficial cells while reducing the growth of regulatory or inflammatory subsets. These strategies must be refined for each tumor target because the tumor response to inflammation is not uniform across tumor types.

We know that many, but not all tumors have the capacity for activating Vγ9Vδ2 T cells. Perhaps the best-studied example is the Daudi Burkitt’s lymphoma cell. The Vγ9Vδ2 T cells are stimulated by contact with Daudi B cells (9) but not by the related Raji cells (10). Activated Vγ9Vδ2 T cells killed Daudi, Raji, and four other Burkitt’s lines including HH514, DG75, Ramos, and Wilson, along with freshly isolated primary tumor cells (10). Subsequently, oligodendrocytes, fetal astrocytes, and glial cells were shown to induce Vγ9Vδ2 T cell activation and proliferation (77), and this activity was correlated with cell surface expression of heat shock proteins. An earlier report had noted the stimulatory properties of Daudi cells expressing a GROEL homolog of heat shock protein (9).

The list of tumors killed by Vγ9Vδ2 T cells is longer than the list of tumors capable of activating Vγ9Vδ2 T cells. Perhaps this is telling us that poor activation *in situ* is a mechanism for tumors to evade Vγ9Vδ2 T cell surveillance. Discrepancies in the literature, wherein *in vitro*-activated Vγ9Vδ2 cells kill tumors even if they express PD-1 ligands, may reveal that activation by direct exposure to tumor cells during normal immune surveillance is not strong enough to drive Vγ9Vδ2 T cell eradication of the tumor or to overcome inhibitory costimulation. When Vγ9Vδ2 T cells are activated by phosphoantigen or aminobisphosphonate and the culture medium includes IL-2, IL-15, or other cytokines, the resulting cells demonstrate potent tumor cell cytotoxicity. To date, we have only limited knowledge about Vγ9Vδ2 T cell activation *in vivo*, with or without stimulatory compounds and cytokines. We also know that some tumors may upregulate expression of FDPS, the enzyme responsible for converting IPP to farnesol (78). Such tumors may have smaller pools of IPP hence lesser capacity for directly activating Vγ9Vδ2 T cells. Tumors with upregulated FDPS are also insensitive to cytostatic effects of aminobisphosphonates because excess enzyme overcomes the drug’s competitive inhibition of FDPS (79).

Importantly, tumors may not always be immunogenic in the conventional sense of activating naïve αβ T cells but may still be targets for Vγ9Vδ2 T cells. Downregulation of MHC is unlikely to affect Vγ9Vδ2 T cell recognition either for activation or as a target for cytolysis and might make tumors more sensitive to killing. Daudi, for example, is a potent activator of Vγ9Vδ2 T cells and a sensitive target despite having no detectable MHC class I expression due to a deletion in the beta2-microglobulin gene (9).

We also note the potential for modulating tumor immunity through specific costimulation of Vγ9Vδ2 T cells. We initially discovered the important role for costimulatory Vγ9Vδ2 T cells in activating tumor cytotoxicity by NK cells (80). Transient expression of 4-1BB (CD137) after phosphoantigen or aminobisphosphonate stimulation of blood Vγ9Vδ2 T cells upregulated this costimulatory molecule, increased NKG2D expression on NK cells, and increased NK tumor effector function. Subsequently, we discovered that Vγ9Vδ2 T cells also signal NK cells through an ICOS:ICOS-L interaction resulting in increased CD69 and 4-1BB expression on NK cells and increased levels of interferon-γ, TNF-α, MIP-1β, 1-309, RANTES, and soluble FasL in the culture. Perhaps most importantly, NK cells “educated” through the ICOS:ICOS-L pathway by Vγ9Vδ2 T cells gained the capacity to kill mature dendritic cells (81). Removing these dendritic cells would alter the tumor microenvironment by reducing inflammation. We also know that NK cells are normally responsible for “licensing” the dendritic cell population primarily by killing immature dendritic cells [reviewed in Ref. (82, 83)]. Both NK and Vγ9Vδ2 T cells interact reciprocally with dendritic cells (84–86) and examples cited earlier (81) showed that activated Vγ9Vδ2 T cells educate NK, which then destroy mature dendritic cells that would normally promote inflammation and tumor growth. Dendritic cells infected by *Brucella melitensis* are substantially impaired in their capacity for antigen presentation but the defect was corrected through a contact-dependent interaction with Vγ9Vδ2 T cells (87). This triangle of Vγ9Vδ2 T cells:NK cells:dendritic cells is part of a regulatory network affecting tumor cell cytotoxicity and regulating inflammation in the tumor microenvironment. We might imagine that mesenchymal stem cells, tissue macrophages, myeloid-derived suppressor cells, and potentially neutrophils or other inflammatory cells have similarly complex interactions that balance the requirement for activating protective immunity with a mechanism to limit destructive inflammation. Understanding these subtle interactions and finding ways to manipulate the regulatory networks may be one key to potent tumor immunotherapies focused on γδ T cells.

The Vγ9Vδ2 subset of γδ T cells is uniquely adapted for tumor immunity through: non-reliance on MHC expression, relative insensitivity to PD-1 inhibition, potent and broad tumor cytotoxicity, low contribution to IL-17A production, activation of NK cytotoxicity, and costimulation of NK for killing of mature (inflammatory) dendritic cells. Several of these mechanisms are unique to Vγ9Vδ2 T cells and fill critical gaps in tumor immunotherapy that are not approached through CAR-T cell therapy or use of checkpoint inhibitor antibodies. By careful selection of appropriate tumor types and understanding the critical markers signaling a healthy versus unhealthy balance of γδ T cell subsets, we can exploit the natural properties of γδ T cells and overcome several well-known mechanisms for tumor evasion of host immunity.

## Preclinical and Clinical Studies of Vγ9Vδ2 T Cell Therapy

Activated Vγ9Vδ2 T cells kill a broad range of tumor cell lines, often with spectacular potency. Investigators have even observed potent killing with effector to target cell ratios below 1, meaning the effector cells recycle without being killed themselves or soluble death ligands are important contributors to cytotoxicity. Several preclinical and clinical studies have tested whether this level of potency translates to potent therapeutic effects *in vivo*.

The SCID mouse model was used to test the tumor surveillance capacity of Vγ9Vδ2 T cells. Mice were injected with Daudi cells followed by injecting PBMC from healthy adult donors. The Daudi cells were sufficient to stimulate Vγ9Vδ2 T cells, resulting in proliferation and transition to effector memory phenotype, along with suppression of tumor growth and survival of the mice (88). Subsequently, tumor killing by Vγ9Vδ2 T cells was demonstrated in several types of immune-deficient mice and with a variety of tumors including prostate cancer (89), melanoma (90), breast cancer (91, 92), ovarian cancer (93), and lymphoma (56, 94, 95) to name a few examples from this growing list.

Mouse xenograft studies demonstrated the potency of Vγ9Vδ2 therapy *in vivo* and the range of tumors that might be treated. In general, treatments were most successful when Vγ9Vδ2 cells were expanded *ex vivo* prior to injection, when cell treatments coincided with tumor cell implantation or occurred when tumors were first deemed “palpable” (meaning < 100 mm^3^ volume) and required repeated administration of phosphoantigen or aminobisphosphonate drugs plus cytokine (usually IL-2). The mouse xenograft studies provided some assurance that Vγ9Vδ2 cell therapy might be successful, but solid proof-of-concept data will be difficult to obtain in this system and safety studies needed for regulatory approval will be challenging. Because human Vγ9Vδ2 T cells are exquisitely species-restricted, normal mouse tissues are not recognized and off-target effects may be obscured. The mouse studies mimic a treatment approach based on adoptive cell therapy but are less useful for studying primary Vγ9Vδ2 tumor responses. In this regard, it is important to note that most clinical studies were completed without serious adverse events.

Mouse model studies raised intriguing issues related to Vγ9Vδ2 T cell trafficking and tumor localization. Knowing that the circulating pool of Vγ9Vδ2 T cells contains both central and effector memory cells, it seems reasonable that at least the effector memory subset would be actively attacking tumors. Injecting Vγ9Vδ2 T cells without additional treatment failed to demonstrate tumor-infiltrating cells and failed to reduce tumor volume. Treating myeloma patients with zoledronic acid increased Vγ9Vδ2 T cell migration into the tumor and infiltration depended on IPP secretion (96). An earlier study on mouse Vγ9Vδ2 T cell migration into murine tumors used antibody blocking to show a requirement for T cell receptor in chemotaxis and tumor infiltration (91). By a mechanism that is not yet established, elevated IPP levels and T cell receptor-dependent mechanisms are associated with Vγ9Vδ2 T cell infiltration into tumors, which is exaggerated by aminobisphosphonate treatment and the resulting increases in IPP levels. Such observations are important, but mechanistic insight into tumor infiltration by phosphoantigen-specific Vγ9Vδ2 T cells is still lacking. It is very important to understand γδ T cell trafficking and mechanisms controlling tumor infiltration.

Human clinical trials have created the greatest promise for Vγ9Vδ2 T cell immunotherapy but also revealed important obstacles to success. The limited potency of γδ T cell immunotherapy is the most pressing problem. Potency is a critical parameter even early in clinical product development, because Vγ9Vδ2 T cell therapies will be compared to results from CAR-T treatments for lymphoma and myeloma. The spectacular cure rates for CAR-T in selected diseases have raised expectations among scientists, patients, advocates, and funders. Positive results from Vγ9Vδ2 therapy in Hodgkin’s lymphoma or multiple myeloma clinical trials showed significant *in vivo* activation of Vγ9Vδ2 T cells among 55% of patients who were pre-screened for high *in vitro* responses to pamidronate/IL-2, along with objective clinical responses among 33% of the pre-screened patients (97). Viewed objectively, these outcomes do not compare favorably with high cure rates for CAR-T in similar diseases (98). Undoubtedly, γδ-centric immunotherapy is eventually less complex and probably safer than CAR-T because the T cells are not genetically modified, but differences in potency will impede both research and commercial development of γδ-centric therapeutics until more data are available.

Tumor immunotherapy with Vγ9Vδ2 T cells, including *in vivo* stimulation, adoptive transfer of expanded cells or combination protocols, may find better purchase in solid tumor treatments where CAR-T is less advanced (98). Immunotherapy based on Vγ9Vδ2 T cells is not limited to tumors with well-defined neoantigens and allogeneic cell products may be possible due to the MHC-unrestricted responses of Vγ9Vδ2 T cells. Treatments based on Vγ9Vδ2 T cells have been tested for head and neck cancer (99), renal carcinoma (100, 101), prostate cancer (102), neuroblastoma (103), mammary carcinoma (104), and lung cancer (105, 106) among others. In most cases, objective responses were noted but the proportion of complete remissions was low and long-term disease-free survival data are minimal. These clinical studies provided evidence for the clinical utility of therapies aimed at activating the tumor response of Vγ9Vδ2 T cells. Clinical and basic researchers in this field need to chart a course for improving these therapies in terms of potency and defining the mechanism of action. We need to understand conditions controlling tumor infiltration by γδ T cells, how cytotoxic and regulatory subsets are regulated, and to understand failures or examples of low potency. This is a complex field with many different approaches and emphases that cannot be covered here in sufficient detail. The reader is encouraged to access several excellent reviews of clinically relevant studies that provide additional examples and important insights into trial outcomes and future directions (48, 49, 51, 107, 108).

## Finding Solid Ground

Researchers in this field are searching for ways to achieve more impactful and curative γδ T cell immunotherapies. If we can realize the full potential of tumor surveillance by these cells, it will be possible to address malignant disease in a broader part of the population than can be reached by other I/O approaches. Several studies are already pointing to more potent strategies. When Vγ9Vδ2 T cells, aminobisphosphonate, and IL-2 were delivered intratumorally in a murine xenotransplant model for glioblastoma, potent tumor reduction was observed (109). Why was intratumoral delivery better than systemic delivery of aminobisphosphonate? Aminobisphosphonate drugs are tremendous for their intended purpose of treating osteoporosis but have unfavorable pharmacokinetics because they complex with calcium and precipitate in the bone matrix. Although the drug may remain in bone for 10 years or more, circulating aminobisphosphonate is eliminated rapidly by renal excretion (110). Rapid clearance of aminobisphosphonates from plasma impedes their use for tumor therapy except in special cases where accumulation in bone was related to the anti-myeloma activity of pamidronate (111). Direct intratumoral injection of aminobisphosphonate avoided the unfavorable pharmacology of this drug class. Poor systemic availability of aminobisphosphonate drugs accounts in part, for differences between extraordinary Vγ9Vδ2 tumor killing *in vitro* where there is no bone to trap the drug, and the lower potencies observed in clinical trials. One of the possible keys to exploiting Vγ9Vδ2 T cells for tumor therapy is to activate them locally and achieve higher potency. In many cases, it may be difficult to continue repeated injections of cells, aminobisphosphonate, and cytokine as was done for glioblastoma in a murine model (109), but studies of this type are beginning to highlight the potential benefits for local activation of Vγ9Vδ2 T cells.

We also know that aminobisphosphonate drugs are competitive inhibitors of FDPS. The FDPS is upregulated in some cancers (112) resulting in resistance to aminobisphosphonate drugs (113). Drug resistance was reversed *in vitro* by transfecting small inhibitory RNA targeting the FDPS mRNA to lower the enzyme levels (113). Thus, we expect an inverse relationship between levels of the stimulatory phosphoantigen IPP and levels of FDPS; reducing FDPS through genetic manipulation is a strategy for increasing the levels of IPP and may be combined with aminobisphosphonate for even higher potency. Lentivirus vector delivery of shRNA targeting FDPS mRNA was tested *in vitro*. The B cell lymphoma line Raji, a poor stimulator of Vγ9Vδ2 cell proliferation or effector function, was transduced with lentivirus vector expressing shRNA against FDPS mRNA. The modified cells were cultured with primary Vγ9Vδ2 T cells to detect changes in phenotype or function. Transduction reduced FDPS levels and markedly activated Vγ9Vδ2 T cells cocultured with the modified Raji cells. Raji cells with decreased FDPS also had increased sensitivity to Vγ9Vδ2 cytotoxicity (114).

Locally administered cancer therapies are increasingly of interest for stimulating potent tumor immunity. Peritumoral, intratumoral, and intranodal therapies have already been tested for a variety of cancers. Viral vectors expressing cytokines, tumor targeting antibodies, and checkpoint inhibitor antibodies appear to be more potent when injected into the tumor microenvironment as opposed to systemic delivery. Intratumoral injection of checkpoint inhibitor antibodies was explored as a means for improving potency while reducing the toxicity encountered after systemic administration [reviewed in Ref. (115)]. Simultaneous intranodal administration of the tumor-targeting antibody Rituximab plus autologous dendritic cells plus granulocyte-macrophage colony stimulating factor caused objective clinical responses in 36% of patients with disseminated follicular lymphoma, an aggressive disease with no known cure (116). Intranodal injection of an adenovirus vector expressing CD40L (CD154) caused objective responses to chronic lymphocytic leukemia in 11 of 15 patients treated (117). Intranodal injection may be viewed as a way to localize therapy near a tumor mass, that also allows stimulation of the immune cells outside of the immunosuppressive tumor microenvironment. These and similar findings encourage us to consider whether potency of Vγ9Vδ2 T cell immunotherapy for cancer has been limited by efforts to stimulate cells through systemic administration of drugs and cytokines, especially when using aminobisphosphonate drugs with unfavorable pharmacokinetics? It seems possible, even likely, that treatment potency will increase dramatically once we achieve potent delivery of stimulating agents to the tumor itself, either by intratumoral or intranodal delivery. Local activation of Vγ9Vδ2 T cells that will infiltrate the tumor, may come closer to realizing the full potential of these innate-like T cells for attacking a broad range of cancers without genetic manipulation of lymphocytes themselves.

## Summary

The remarkable γδ T cells continue to be a focus for the development of new cancer immunotherapies because they are an important component of natural tumor surveillance. The Vγ9Vδ2 T cells are particularly attractive for tumor therapy because they comprise the largest group of memory T cells responding to a single antigen. These phosphoantigen-responsive cells represent about 2% of total T cell memory in the circulating population and respond with the speed and uniformity of innate immunity. Basic and clinical research on Vγ9Vδ2 T cells and other γδ T cell subsets is already demonstrating utility in cancer therapy, but the challenge is to increase potency and understand better the mechanisms of action. The improvements in patient outcomes will come through better definition of the balance between effector and regulatory subsets, the role for inhibitory costimulation pathways, factors governing tumor infiltration, and methods for increasing potency. The keys to potency and tumor elimination may be found in local administration of stimulating agents including chemicals, cytokines, and viral vectors. All of these obstacles pale in comparison to the value of an immunotherapy that attacks a broad range of tumor cell types, does not require identification of tumor-associated antigens, and does not require genetic modification of T cells. Local treatment delivered at the tumor site may be one way to increase Vγ9Vδ2 T cell potency. The promise is to achieve a near universal solution for malignant disease. The challenge is to translate the exquisite science of γδ T cell biology for the practical goal of cancer immunotherapy.

## Author Contributions

All authors listed have made a substantial, direct, and intellectual contribution to the work and approved it for publication.

## Conflict of Interest Statement

CP, M-LL, TL, LX, and HL are full time employees of American Gene Technologies Intnl. (AGT), Inc. in Rockville, MD. AGT has early stage research and development programs in gamma delta T cell immunotherapy for cancer focusing on the use of lentiviral vectors for manipulating tumor cells. AGT has received a patent on viral vector modification of cancer cells for activating Vg9Vd2 T cells (US 9,834,790) and is supported by private investment related partly to the immuno-oncology program. CC, PhD and RL, PhD are full time employees of the University of Maryland School of Medicine. CC is independently funded (NIH/NIAID) for research on pediatric gamma delta T cells in infectious disease. RL is Director of Translational Sciences in the Greenebaum Comprehensive Cancer Center at the University of Maryland School of Medicine. She is funded through a grant from AGT to perform cancer therapy studies in mouse models. RL receives no personal remuneration from AGT including no personal fees and no stock grants. She does not share in intellectual property of the company and is a co-author of this review because of her deep understanding of animal models for cancer therapy.
